# Prevalence of musculoskeletal disorders among school teachers from urban and rural areas in Chuquisaca, Bolivia: a cross-sectional study

**DOI:** 10.1186/s12891-017-1785-9

**Published:** 2017-10-27

**Authors:** María Teresa Solis-Soto, Anabel Schön, Angel Solis-Soto, Manuel Parra, Katja Radon

**Affiliations:** 1 Universidad San Francisco Xavier de Chuquisaca, Estudiantes, 96 Sucre, Bolivia; 2 Institute for Occupational, Social and Environmental Medicine, Occupational and Environmental Epidemiology & Net Teaching Unit, University Hospital Munich (LMU), Munich. Ziemssenstr. 1, 80336 Munich, Germany; 3 Centro de Diagnóstico Neurológico, Urriolagoitia, 354 Sucre, Bolivia

**Keywords:** Musculoskeletal disorders, Nordic questionnaire, Rural, Schools teachers, Urban

## Abstract

**Background:**

Musculoskeletal disorders (MSD) are important health problems in working populations. The study aimed to determine the prevalence of MSD among school teachers from urban and rural areas in Chuquisaca, Bolivia.

**Methods:**

A cross-sectional study was conducted in 60 randomly selected schools. In total, 1062 teachers were invited to participate (response 58%). The Spanish version of the Standardized Nordic questionnaire was used assessing the 12-months and 7-days prevalence of MSD as well as the 12-months prevalence of work limiting pain. Prevalence were calculated for the different parts of the body; as summary measures, MSD in any part of the body and in ≥3 parts of the body were assessed. Crude and adjusted odds ratios with 95% confidence intervals were calculated using logistic regression models adjusting for age, sex, teaching level and school type.

**Results:**

Prevalence of MSD in any part of the body was 86% during the last 12 months, 63% during the last 7 days and 15% for work limiting pain. MSD was most common in the neck (12-months prevalence 47%) and least common in the wrist/hands (26%). In the adjusted model, teachers working in rural areas presented significantly higher odds than teachers from urban schools for work-limiting pain during the last 12-months considering any part of the body (aOR 2.2; 95% CI 1.1–4.1), and for ≥3 parts of the body (aOR 3.7; 95% CI 1.3–10.6).

**Conclusion:**

The prevalence of MSD is high in School teachers, even more in teachers working in rural areas. It is needed to identify risk factors for MSD in teachers in order to propose appropriate strategies to control and reduce it.

**Electronic supplementary material:**

The online version of this article (10.1186/s12891-017-1785-9) contains supplementary material, which is available to authorized users.

## Background

Musculoskeletal disorders (MSD) have been reported as one of the most common and important health problems in working populations, generating social and economic implications [1]. In teachers, prevalence of MSD was found to range between 39% and 95% [2]. In general, they reported more frequently back, neck and upper limbs problems although there have been marked differences between the type of teacher and the body part affected.

Teachers working conditions in rural schools face greater challenges than in urban areas, such as social and geographic isolation, poor working conditions, poor remuneration, limited opportunities for professional improvement, lack of adequate resources, careless buildings, cultural differences and poor community involvement [3, 4]. Additionally educational system in many countries has no adequate strategies to attract and retain competent and qualified teachers [5], impacting on working conditions with greater limitations in rural schools.

Some studies reported that physical factors such as prolonged standing, sitting and uncomfortable posture are known to be associated with increased prevalence of MSD [6]. In addition, several studies suggest that psychosocial factors including high workload and demands, high perceived stress levels, low social support, low job control, low job satisfaction and monotonous work are associated with MSD among school teachers [7]. In this sense, it is possible that teachers in rural areas may be more exposed to develop musculoskeletal disorders.

In Bolivia, teachers and administrative staff working in the educational sector represent the largest public workforce (around 100,000 workers) [8]. According to the World Bank, expenditure of the Bolivian Government on education increased from 5.7% in 1999, to 6.4 in 2012 [9]. Chuquisaca, one of the nine departments (regions) in Bolivia, is located in the south part of the country. It has an estimated population of 616,073 inhabitants from which 50.2% are living in rural areas [10]. Illiteracy rate in Chuquisaca is around 8 to 37%, school attendance between 69 to 89% and coverage for primary education 86.2%, lower than the national coverage (92%) [11]. As in other Latin American countries, educational indicators in Bolivia and in Chuquisaca show big differences between urban and rural areas with lower educational levels and more unfavorable working conditions in rural areas [11–13].

Working and health conditions in Latin-American and especially in Bolivian teachers have been scarcely explored. In the same way, there are few reports exploring health conditions in teachers working in rural areas. Therefore the objectives in our study were to determine the prevalence of musculoskeletal disorders considering the whole body and to compare differences between schools teachers working in rural and urban areas of Chuquisaca, Bolivia.

## Methods

A cross-sectional study was conducted in Chuquisaca, Bolivia from August to November 2015.

### Participants

The Bolivian educational system is organized in alternative education (adult, permanent and special education) and regular education which includes preschool, primary, secondary and higher education in public, private and semi-public schools (Public using private infrastructure). Teachers working in regular education in Chuquisaca region were the target group for this study comprising a total of 8954 teachers working in 1284 schools (Regional Education Direction Register 2014), 57% of them in rural areas.

In order to sample the teachers, a simple one-stage cluster sampling was performed including all 626 geographically accessible schools with at least 5 teachers. These schools employed about 86% of all teachers in Chuquisaca. Of these, we randomly selected 27 schools located in urban areas and 33 located in rural areas as primary sampling unit and invited all teachers working in those schools (1062) to participate in the questionnaire study.

Sample size was calculated using StatCalc-EpiInfo. An estimated prevalence for 12-months MSD of 50% was considered. In order to reach a statistical power of 80% to detect differences of 5% in the prevalence of MSD between urban and rural areas, 240 teachers in both urban and rural areas were needed.

### Instruments

Validated questions to explore sociodemographic variables and working conditions at school were used from the VI National Survey of Working Conditions and Health (Spain) [14] and from the Working Conditions and Health Teaching Study published by the Regional Office of Education of UNESCO for Latin America and the Caribbean, OREALC/UNESCO respectively [15]. For the assessment of musculoskeletal disorders the Spanish version of the Standardized Nordic questionnaire was used [16]. It explores the 12-months, 7-days prevalence of musculoskeletal disorders or discomfort in neck, wrist or hands, shoulders, upper and low back, hips or thighs, knees and ankles or feet as well as the 12-months prevalence of work limiting pain in the same body areas. This questionnaire has been reported to be a reliable and valid screening and surveillance tool for musculoskeletal disorders (MSD) [17]. An English version of the questionnaire is available in Additional file 1.

Sociodemographic information included: age (categorized into 4 groups: < 30 years, 30–39, 40–49 and ≥ 50 years), gender (male and female), and school location (rural and urban). Teaching level was grouped in exclusive primary teachers, exclusive secondary teachers and teachers working at primary and secondary level. Type of school was categorized in Public and Private (including public school with private infrastructure) considering the type of school administration where the teacher was working most hours.

To define the outcome, the following definitions were used:

- *12-months musculoskeletal disorder (MSD):* was considered present if self-reported ache, pain or discomfort in neck, wrist or hands, shoulders, upper and low back, hips or thighs, knees and ankles or feet during the last 12 months was reported (Separately for each part of the body)
- *7-days MSD:* was defined as 7-days prevalence of MSD.
- *12-months work limiting pain* was defined as work impediment in the last 12 months prior to survey because of MSD.

*Any MSD* was considered when the teacher reported 12-months MSD in any part of the body (neck, wrist or hands, shoulders, upper and low back, hips or thighs, knees and ankles or feet) respectively. If no disorder was present in the last 12-months than also no disorder can be present in the last 7 days nor can the pain be work-limiting. The choice of the time frames was determined based in previous studies in order to explore past pain (during the last 12 months) and also current but chronic or recurrent pain (disorder reported during the last 12 months and during the last 7 days) [16, 18].

The number of the parts of the body affected was computed adding up the number of places that were reported with discomfort. The range was between 0 (no body parts affected) to 8 (all body parts affected). The median value of this sum was used as cut off (≥ 3).

### Procedure

The selected schools were contacted to explain the research objectives and coordinate visits. For every teacher, every school received an envelope with the questionnaire, information sheet and informed consent form. Two boxes were set up in each school, the first one to deposit the full questionnaire (in a sealed envelope), and the second one to deposit the signed informed consent form. The ID number on the questionnaire was not written on the informed consent form in order to ensure anonymity of the study. Teachers were told to note down their personal ID so that they could withdraw participation at any time.

### Data analysis

As quality control a double-entry of data and congruence checking was performed EpiInfo v. 7 for Windows. Data was exported to SPSS v.17 for further analysis.

In order to check for the potential dependence of samples due to the cluster sampling, intraclass correlation coefficients (ICC) were computed to analyze the correlation of responses within the clusters (schools) for each of the outcomes [19]. ICC was less than 0.001 for all definitions of MSD. For that reason the analyses were not adjusted for cluster sampling.

The number of cases and period prevalence of the MSD outcomes were calculated for a general description of the study population comparing the distribution in rural and urban areas. In addition, crude (OR) and adjusted odds ratios (aOR) as well as their corresponding 95% confidence intervals (95%CI) were calculated using bivariate and multivariate logistic regression models. The models were adjusted for sociodemographic and working conditions variables which were statistically significant associated with exposure and outcome (*p* value ≤0.05) in the bivariate analysis (Table 1).

**Table 1:** Description of the study population (*N* = 517)

		Urban n (%)	Rural n (%)	*P* value*
Age (years)	< 30	25 (11.6)	67 (22.6)	< 0.01
	30–39	49 (22.7)	120 (40.5)	
	40–49	61 (28.2)	65 (22.0)	
	≥ 50	81 (37.5)	44 (14.9)	
Gender	Female	166 (76.5)	201 (68.4)	0.04
	Male	51 (23.5)	93 (31.6)	
Teaching level	Primary	120 (55.6)	124 (42.2)	< 0.01
	Secondary	60 (27.8)	134 (45.6)	
	Primary and secondary	36 (16.7)	36 (12.2)	
Type of school	Public	133 (62.7)	276 (93.2)	< 0.01
	Private	79 (37.3)	20 (6.8)	

Some percentages do not add up to 100% due to rounding

*Chi square Test

## Results

A total of 620 teachers returned the complete questionnaire reaching response of 58% (57% in urban and 59% in rural schools). For analysis, 103 questionnaires were excluded because participants were administrative staff (*N* = 19) or did only complete the questionnaire partially (*N* = 84). There were no statistically significant differences in descriptive variables between included and excluded teachers.

In rural areas compared to urban areas, teachers were statistically significantly younger, more likely to be male, to work at the secondary level and to work at a public school (Table 1).

The period prevalence of MSD in any region was 86%, 63% and 15% for the last 12-months, 7-days and 12-months prevalence of work limiting pain respectively and 48%, 26% and 5% for musculoskeletal disorders in three or more parts of the body (Table 2). In general, rural area showed a higher prevalence of MSD although the difference was not statistically significant for all outcome definitions.

**Table 2:** Prevalence of musculoskeletal disorders (MSD) in school teachers from urban and rural areas in Chuquisaca, Bolivia (*N* = 517)

N missing = 7	12-months prevalence			7-days prevalence			12-months prevalence of work limiting pain		
	Total n (%)	Urban n (%)	Rural n (%)	Total n (%)	Urban n (%)	Rural n (%)	Total n (%)	Urban n (%)	Rural n (%)
Neck/Upper extremities									
Neck	244 (47.2)	106 (48.2)	138 (46.5)	158 (30.6)	62 (28.2)	96 (32.3)	31 (6.0)	6 (2.7)	25 (8.4)
Shoulders	179 (34.6)	69 (31.4)	110 (37.0)	92 (17.8)	30 (13.6)	62 (20.9)	18 (3.5)	3 (1.4)	15 (5.1)
Wrist/Hands	133 (25.7)	47 (21.4)	86 (29.0)	72 (13.9)	24 (10.9)	48 (16.2)	14 (2.7)	4 (1.8)	10 (3.4)
Any MSD in upper extremities	325 (63.7)	134 (62.0)	191 (65.0)	214 (42.0)	81 (37.5)	133 (45.2)	42 (8.2)	8 (3.7)	34 (11.6)
Back									
Upper back	185 (35.8)	68 (30.9)	117 (39.4)	101 (19.5)	37 (16.8)	64 (21.5)	28 (5.4)	8 (3.6)	20 (6.7)
Low back	171 (33.1)	73 (33.2)	98 (33.0)	110 (21.3)	42 (19.1)	68 (22.9)	27 (5.2)	7 (3.2)	20 (6.7)
Any back disorder	238 (46.7)	95 (44.0)	143 (48.6)	142 (27.8)	56 (25.9)	86 (29.3)	37 (7.3)	10 (4.6)	27 (9.2)
Lower extremities									
Hips/Thighs^a^	165 (31.9)	65 (29.5)	100 (33.7)	101 (19.5)	41 (18.6)	60 (20.2)	19 (3.7)	4 (1.8)	15 (5.1)
Knees^a^	194 (37.5)	86 (39.1)	108 (36.4)	117 (22.6)	46 (20.9)	71 (23.9)	25 (4.8)	8 (3.6)	17 (5.7)
Ankles/Feet^a^	157 (30.4)	51 (23.2)	106 (35.7)	100 (19.3)	36 (16.4)	64 (21.5)	19 (3.7)	5 (2.3)	14 (4.7)
Any disorder in lower extremities	312 (61.2)	127 (58.8)	185 (62.9)	190 (37.3)	78 (36.1)	112 (38.1)	39 (7.6)	10 (4.6)	29 (9.9)
Summary measures: Any part of the body									
Any MSD^b^	442 (85.5)	186 (84.5)	256 (86.2)	328 (63.4)	134 (60.9)	194 (65.3)	77 (14.9)	19 (8.6)	58 (19.5)
MSD in ≥ 3 parts of the body	247 (47.8)	98 (44.5)	149 (50.2)	132 (25.5)	50 (22.7)	82 (27.6)	25 (4.8)	6 (2.7)	19 (6.4)

^a^One or both

^b^MSD in neck, shoulders, wrist, hands, upper or lower back, hips, thighs, knees, ankles or feet

The 12-months prevalence of musculoskeletal disorders ranged from 26% for wrist/hands to 47% for neck. Only for neck and knee disorders the prevalence was higher in urban areas. Following the same pattern, 7-day prevalence ranged from 14% for disorders in wrist/hands to 31% for neck disorders. For work limiting pain during last 12-months it ranged from 3% for wrist/hands to 6% for neck (Table 2).

The adjusted logistic regression models basically confirmed the bivariate analyses: teachers working in rural areas reported statistically significantly higher odds than teachers from urban schools for any work limiting musculoskeletal pain during the last 12-months prior to survey (aOR 2.2; 95% CI 1.1–4.1) and for work limiting pain in at least 3 parts of the body (aOR 3.7; 95% CI 1.3–10.6) (Table 3). For specific parts of the body, work-limiting pain in ankles or feet was higher in rural than in urban teachers (aOR 4.4; 95% CI 1.4–13.7). No statistically significant differences were seen for the 12-months nor the 7-days prevalence of symptoms regardless of body part in these adjusted models.

**Table 3:** Unadjusted and adjusted odds ratios comparing the prevalence of musculoskeletal disorders (MSD) in Bolivian school teachers from urban and rural areas (*N* = 517)

N missing = 23	12-months prevalence		7-days prevalence		12-months work limiting pain prevalence	
	Crude OR^a^ (95% CI)	Adjusted OR^a,b^ (95% CI)	Crude OR^a^ (95% CI)	Adjusted OR^a,b^ (95% CI)	Crude OR^a^ (95% CI)	Adjusted OR^a,b^ (95% CI)
Neck/Upper extremities						
Neck	0.93 (0.7–1.3)	0.65 (0.4–1.0)	1.22 (0.8–1.8)	0.84 (0.5–1.3)	3.28 (1.3–8.1)	2.59 (0.9–7.5)
Shoulders	1.29 (0.9–1.9)	1.23 (0.8–1.9)	1.67 (1.0–2.7)	1.81 (1.0–3.2)	3.85 (1.1–13.5)	3.59 (0.9–14.8)
Wrist/Hands	1.50 (1.0–2.3)	1.36 (0.8–2.2)	1.58 (0.9–2.7)	1.29 (0.7–2.4)	1.88 (0.6–6.1)	1.83 (0.4–7.5)
Any MSDin upper extremities	1.14 (0.8–1.6)	0.87 (0.6–1.4)	1.38 (1.0–2.0)	1.15 (0.7–1.8)	3.40 (1.5–7.5)	2.51 (1.0–6.3)
Back						
Upper back	1.45 (1.0–2.1)	1.15 (0.7–1.8)	1.36 (0.9–2.1)	1.02 (0.6–1.7)	1.90 (0.8–4.4)	2.26 (0.8–6.1)
Low back	0.99 (0.7–1.4)	0.77 (0.5–1.2)	1.26 (0.8–1.9)	0.77 (0.5–1.3)	2.20 (0.9–5.3)	1.63 (0.6–4.6)
Any back disorder	1.21 (0.8–1.7)	1.08 (0.7–1.6)	1.18 (0.8–1.8)	0.87 (0.5–1.4)	2.08 (1.0–4.4)	2.06 (0.9–4.9)
Lower extremities						
Hips/Thighs (One or both)	1.21 (0.8–1.8)	0.96 (0.6–1.5)	1.11 (0.7–1.7)	0.84 (0.5–1.4)	2.87 (0.9–8.8)	2.64 (0.8–9.2)
Knees (One or both)	0.89 (0.6–1.3)	1.07 (0.7–1.6)	1.19 (0.8–1.8)	1.21 (0.7–2.0)	1.60 (0.7–3.8)	2.04 (0.8–5.3)
Ankles/Feet (One or both)	1.84 (1.2–2.7)	2.25 (1.4–3.6)	1.40 (0.9–2.2)	1.60 (0.9–2.7)	2.13 (0.8–6.0)	4.35 (1.4–13.7)
Any MSD in lower extremities	1.18 (0.8–1.7)	1.27 (0.8–1.9)	1.09 (0.8–1.6)	0.95 (0.6–1.5)	2.25 (1.1–4.7)	2.17 (0.9–5.0)
Summary measures: Any part of the body						
Any MSD	1.14 (0.7–1.9)	1.14 (0.6–2.1)	1.21 (0.8–1.7)	0.96 (0.6–1.5)	2.57 (1.5–4.5)	2.16 (1.1–4.1)
MSD in ≥ 3 body places	1.25(0.8–1.8)	1.00 (0.7–1.5)	1.30 (0.9–1.9)	0.95 (0.6–1.5)	2.44 (1.0–6.2)	3.69 (1.3–10.6)

^a^Reference category: Urban area (OR = 1)

^b^Adjusted by age, sex, teaching level and school type

## Discussion

This study aimed to explore self-reported musculoskeletal disorders (MSD) considering 12-months and 7-days prevalence as well as the 12-months prevalence of work limiting pain comparing teachers from urban and rural schools. Prevalence of MSD was high and especially work-limiting pain was more common in teachers employed at rural than in those working in urban schools.

Our results showed considerable prevalence of MSD affecting several parts of the body maybe due to the variety of activities that teachers perform each day at work. Almost half of the participants reported MSD in ≥ 3 parts of the body during the last 12-months and 26% during the last 7-days. It is relatively high if we compare with Brazilian teachers, where 7-days prevalence (≥ 3 parts of the body) was 15.9% in 2011 [20]. This could be explained due to differences in geographic context. Brazilian study comprised metropolitan area with high Municipal Human Development Index (0.717) than the region studied in Bolivia (0.563) [21], and probably working and living conditions are different.

In agreement with previous studies, we found considerable prevalence of disorders reported in the neck and upper extremities but also in back and lower extremities [2]. Although the main focus of the present study is to compare the prevalence of musculoskeletal disorder in urban and rural areas, a major limitation of the study is the lack of inclusion of risk factors that explain these differences. Individual factors, as well as working conditions under which the teachers perform their work could explain the presence of specific musculoskeletal disorders for each region of the body. MSD in neck and upper extremities can be a consequence of the significant use of uncomfortable physical activities, like ‘head down’ posture, during reading, writing on a blackboard or marking of assignments for several hours [22, 23]; but also back and lower extremities could be affected due to long hours standing while teaching [24], postural overloads in the classroom, uncomfortable back support while seated, recurrent twisting, and prolonged static postures [25]. Additionally teachers face every day social and psychological demands inside and outside the school [26, 27], and have reported less time for rest after work, because of extra work at home [28], which could lead in chronic and disabling musculoskeletal disorders [29]. Little information is available about work limiting pain prevalence in teachers. Our results reported a prevalence around 15% for any musculoskeletal disorder, lower than previously reported. One study in Brazilian public school teachers found a prevalence of work-limiting pain in any part of the body of 47.4% [30], also Converso et al. reported a prevalence of 42.9% suffering moderate to severe limiting musculoskeletal pain in nursery school and kindergarten teachers in Italy [31]; In the same way staff at special schools in Germany reported a prevalence of chronic back pain of 27.6% [32]. This difference could be explained, because those studies explored MSD mainly in teachers from special schools, where physical demands as frequently carrying and lifting heavy loads must be taken into consideration.

This is the first study comparing working and health conditions in teachers working in urban and rural areas in Bolivia. Our study showed higher MSD prevalence in rural areas, especially for work-limiting pain independently to age, sex, teaching level and school type. Living and working conditions in rural areas of Bolivia, represents a great challenge for professionals, especially because limited geographic access, bad road conditions, distancing from the family and poor social support between peers, limited access to technology (including internet), language and cultural issues and poor academic support of parents [4]. Additionally the perceived role of the teacher in the rural areas implies a closer work with the community which could demand more physical and psychological demands in the daily work [33]. This situation often leads to the concentration of professionals in urban areas impacting on the quality of education and increasing inequalities between these two areas [34].

Most of the studies explored musculoskeletal disorders through self-reported questionnaires. Although this could have its limitations, Nordic Questionnaire has shown to be a good screening tool, especially in occupational settings. A study found a sensitivity of 100% and specificity of 88% to detect subjects with chronic or recurring low back pain for this questionnaire [17]. Additionally information about pain or discomfort during the last seven days could provide more reliable information minimizing memory recall bias. In this sense we may assume that this situation is not different in our study because teachers were able to understand these questions through their level of education, and fill it in a reliable way due to the anonymous report in the study.

Even though our study had 58% of response, it is within the expected percentage considering studies which focused in musculoskeletal disorders in schools teachers [2]. Nevertheless it is possible that teachers who did not want to participate in the study were those with greater workload and possibly those who may have presented less muscular disorders. Due to feasible reasons, we had to exclude schools with very difficult geographic access or with very few students, mainly located in the rural area (less than 15% from the total). Working conditions and in particular psychosocial factors at work could be a challenge for teachers working in those schools because perception of social, cultural, and professional isolation reported in teacher working in rural areas [35]. For that reason it is possible that the differences between rural and urban areas could be underestimated in our study.

## Conclusions

Although prevalence of musculoskeletal disorders is considerable in school teachers in Chuquisaca, it is within the range reported previously. Teachers working in rural areas reported higher prevalence and more severe symptoms than teachers working in urban areas. It is needed to explore in-depth risk factors related to musculoskeletal disorders in this occupational group in order to propose appropriate strategies to control and reduce it. In the same way, it is important to consider surveillance systems in working conditions which include musculoskeletal disorders in teachers.

## Additional file

Additional file 1: Work and health Conditions in Latin America – School teachers Questionnaire. It includes basic pack of questions to assess Musculoskeletal disorders (Nordic Questionnaire) and sociodemographic variables and working conditions at school. (DOCX 651 kb)

## Abbreviations

95%CI: 95% Confidence intervals
aOR: Adjusted odds ratio
ICC: Intraclass correlation coefficients
MSD: Musculoskeletal disorders
OR: Odds ratio
OREALC: Regional Bureau of Education for Latin America and the Caribbean
UNESCO: United Nations Educational, Scientific and Cultural Organization

## References

[1] Summers K, Jinnett K, Bevan S. Musculoskeletal disorders, workforce health and productivity in the United States. The center for workforced health and performance. London: Lancaster university; 2015.

[2] Erick PN, Smith DRA (2011). Systematic review of musculoskeletal disorders among school teachers. BMC Musculoskelet Disord.

[3] Adedeji SO, Olaniyan O (2011). Improving the conditions of teachers and teaching in rural schools across African countries.

[4] Villarroel Rosende G, Sánchez SX. Relación familia y escuela: Un estudio comparativo en la ruralidad. Estudios pedagógicos (Valdivia). 2002;(28):123–41.

[5] Alcalde DEV (2006). Atraer y retener buenos profesionales en la profesión docente: políticas en Latinoamérica. Revista de Educación.

[6] Mohan V, Justine M, Jagannathan M, Bt Aminudin S, Bt Johari SH (2015). Preliminary study of the patterns and physical risk factors of work-related musculoskeletal disorders among academicians in a higher learning institute. J Orthop Sci.

[7] Erick P, Smith D (2013). Musculoskeletal disorder risk factors in the teaching profession: a critical review. OA Musculoskelet Med.

[8] United Nations Educational SaCOU (2010). World data on education - Bolivia.

[9] Bank W. Government expenditure on education, total (% of GDP) [Internet]. 2012 [updated 2016; cited 2017 January, 3]. Available from: http://data.worldbank.org/indicator/SE.XPD.TOTL.GD.ZS.

[10] National Institute of Statistics (INE). Population projections by department and municipality, 2012–2020 [Internet]. 2016 [cited 2016 October, 7]. Available from: http://www.ine.gob.bo/.

[11] National Institute of Statistics Bolivia. Socioeconomic statistics of the department of Chuquisaca [Internet]. 2011 [cited 2017 January, 8]. Available from: http://www.ine.gob.bo/index.php/prensa/publicaciones.

[12] Lopez N, Pereyra A, Sourrouille F. Disparidades urbanas y rurales en América Latina. Algunas de sus implicancias en el acceso a la educación. Buenos Aires, Argentina: United Nations Educational, Scientific and Cultural Organization; 2007.

[13] La YM (2011). educación rural en Chuquisaca. Elementos para futuras investigaciones.

[14] Almodóvar A, Pinilla F (2007). VI National Survey of working conditions (ENCT).

[15] Robalino M, Körner A (2005). Condiciones de trabajo y salud docente. Estudios de casos en Argentina, Chile, Ecuador, México, Perú y Uruguay.

[16] Kuorinka I, Jonsson B, Kilbom A, Vinterberg H, Biering-Sorensen F, Andersson G (1987). Standardised Nordic questionnaires for the analysis of musculoskeletal symptoms. Appl Ergon.

[17] Takekawa KS, Goncalves JS, Moriguchi CS, Coury HJ, Sato Tde O (2015). Can a self-administered questionnaire identify workers with chronic or recurring low back pain?. Ind Health.

[18] Hoy D, Brooks P, Blyth F, Buchbinder R (2010). The epidemiology of low back pain. Best Pract Res Clin Rheumatol.

[19] Killip S, Mahfoud Z, Pearce K (2004). What is an intracluster correlation coefficient? Crucial concepts for primary care researchers. Ann Fam Med.

[20] de Ceballos AG, Santos GB (2015). Factors associated with musculoskeletal pain among teachers: sociodemographics aspects, general health and well-being at work. Rev Bras Epidemiol.

[21] Programa de las Naciones Unidas para el Desarrollo – PNUD. Índice de Desarrollo Humano en los Municipios de Bolivia. Informe Nacional de Desarrollo Humano 2004. La Paz, Bolivia; 2004.

[22] Bogaert I, De Martelaer K, Beutels M, De Ridder K, Zinzen E (2016). Posture analysis among Flemish secondary school teachers: difference between the use of chalkboards and electronic school boards during classroom teaching. Ergonomics.

[23] Chiu TT, Lam PK (2007). The prevalence of and risk factors for neck pain and upper limb pain among secondary school teachers in Hong Kong. J Occup Rehabil.

[24] Abdulmonem A, Hanan A, Elaf A, Haneen T, Jenan A (2014). The prevalence of musculoskeletal pain & its associated factors among female Saudi school teachers. Pak J Med Sci.

[25] Yue P, Liu F, Li L (2012). Neck/shoulder pain and low back pain among school teachers in China, prevalence and risk factors. BMC Public Health.

[26] Arvidsson I, Hakansson C, Karlson B, Bjork J, Persson R (2016). Burnout among Swedish school teachers - a cross-sectional analysis. BMC Public Health.

[27] Agai-Demjaha T, Minov J, Stoleski S, Zafirova B (2015). Stress causing factors among teachers in elementary schools and their relationship with demographic and job characteristics. Open Access Maced J Med Sci.

[28] Shimizu M, Wada K, Wang G, Kawashima M, Yoshino Y, Sakaguchi H (2011). Factors of working conditions and prolonged fatigue among teachers at public elementary and junior high schools. Ind Health.

[29] Vignoli M, Guglielmi D, Balducci C, Bonfiglioli R (2015). Workplace bullying as a risk factor for musculoskeletal disorders: the mediating role of job-related psychological strain. Biomed Res Int.

[30] Fernandes MH, da Rocha VM, Costa-Oliveira d (2009). AG. [factors associated with teachers' osteomuscular symptom prevalence]. Revista de salud publica (Bogota, Colombia).

[31] Converso D, Viotti S, Sottimano I, Cascio V, Guidetti G (2015). Work ability, psycho-physical health, burnout, and age among nursery school and kindergarten teachers: a cross-sectional study. La Medicina del lavoro.

[32] Claus M, Kimbel R, Spahn D, Dudenhoffer S, Rose DM, Letzel S (2014). Prevalence and influencing factors of chronic back pain among staff at special schools with multiple and severely handicapped children in Germany: results of a cross-sectional study. BMC Musculoskelet Disord.

[33] Vera Bachmann D, Osses S, Schiefelbein FE (2012). Las Creencias de los profesores rurales: una tarea pendiente para la investigación educativa. Estudios pedagógicos (Valdivia).

[34] Blanes J (2006). Bolivia: las áreas metropolitanas en perspectiva de desarrollo regional. EURE (Santiago).

[35] Goodpaster KP, Adedokun OA, Weaver GC (2012). Teachers' perceptions of rural STEM teaching: implications for rural teacher retention. Rural Educ.

[36] World Health organization (2011). Standards and operational guidance for ethics review of health-related research with human participants.

