# Supplementary material for: Prevalence of musculoskeletal disorders among school teachers from urban and rural areas in Chuquisaca, Bolivia: a cross-sectional study
Source: BMC Musculoskelet Disord. 2017 Oct 27;18:425. doi: 10.1186/s12891-017-1785-9 (PMC5658995; doi:10.1186/s12891-017-1785-9)
Supplement: Additional file 1: — Work and health Conditions in Latin America – School teachers Questionnaire. It includes basic pack of questions to assess Musculoskeletal disorders (Nordic Questionnaire) and sociodemographic variables and working conditions at school. (DOCX 651 kb) [file 12891_2017_1785_MOESM1_ESM.docx]

| **MAIN MODULE** |
| --- |

| **I have read the study information and the information about the confidentiality of**  **the data and I accept that the data will be used as indicated in the information**  **of the study.** | **Yes** |
| --- | --- |
|  |  |
|  |  |

| **GENERAL QUESTIONS** |
| --- |

**1.- Sex?**

(PG.1)

| Male |  |
| --- | --- |
| Female |  |

**2.- How old are you?**

(PG.2)

| Less than 20 years |  |
| --- | --- |
| 20-29 years |  |
| 30-39 years |  |
| 40-49 years |  |
| 50 years or more than 50 years |  |

**3.- What is your birthplace?**

(PG.3)

| City: |  |  |  |  |  |  |  |  |  |  |  |  |  |  |  |  |  |  |  |  |  |  |
| --- | --- | --- | --- | --- | --- | --- | --- | --- | --- | --- | --- | --- | --- | --- | --- | --- | --- | --- | --- | --- | --- | --- |
|  |  |  |  |  |  |  |  |  |  |  |  |  |  |  |  |  |  |  |  |  |  |  |
| Country: |  |  |  |  |  |  |  |  |  |  |  |  |  |  |  |  |  |  |  |  |  |  |

**4.- What is the highest academic degree achieved?**

| Normal Teacher |  |
| --- | --- |
| University Technician (Middle / Higher) |  |
| Bachelor's degree (University) |  |
| Postgraduate Diploma / Master's / Doctorate |  |

| Other: |  |  |  |  |  |  |  |  |  |  |  |  |  |  |  |  |  |  |  |  |  |  |
| --- | --- | --- | --- | --- | --- | --- | --- | --- | --- | --- | --- | --- | --- | --- | --- | --- | --- | --- | --- | --- | --- | --- |

**5.-** **What type of teacher are you?**

| Primary |  |
| --- | --- |
| Secondary |  |
| Primary and secondary |  |
| Manager |  |

| Other: |  |  |  |  |  |  |  |  |  |  |  |  |  |  |  |  |  |  |  |  |  |  |
| --- | --- | --- | --- | --- | --- | --- | --- | --- | --- | --- | --- | --- | --- | --- | --- | --- | --- | --- | --- | --- | --- | --- |

**6.- Choose the alternative that best reflects your situation regarding the type of educational establishment where you teach (Check all that apply):**

| Public Establishment |  |
| --- | --- |
| Private Establishment |  |
| Establishment of “agreement” |  |


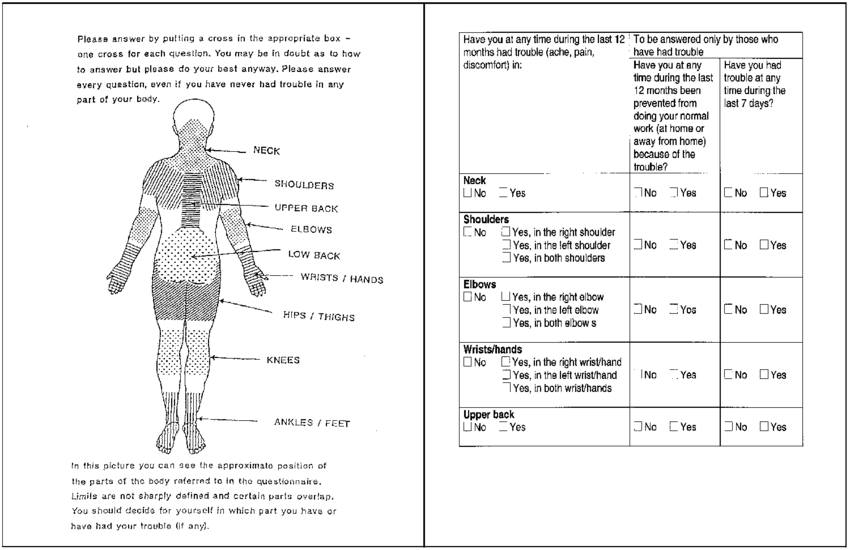


**Module: Musculoskeletal Disorders**

|  | Have you felt discomfort during the last **12 MONTHS** (pain, discomfort / discomfort, numbness) in:  (MM.6) | | | | | If "Yes": Have you had any discomfort during the **last 7 days**?  (MM.7) | | | | | Have you at any time during the last **12 MONTHS** been prevented from doing your job?  (MM.8) | |
| --- | --- | --- | --- | --- | --- | --- | --- | --- | --- | --- | --- | --- |
|  | **NO** | **YES** | **YES, Left** | **YES, Right** | **YES, Both** | **NO** | **YES** | **YES, Left** | **YES, Right** | **YES, Both** | **NO** | **YES** |
| 1. Neck |  |  |  |  |  |  |  |  |  |  |  |  |
| 1. Shoulders |  |  |  |  |  |  |  |  |  |  |  |  |
| 1. Wrists/Hands |  |  |  |  |  |  |  |  |  |  |  |  |
| 1. Upper back |  |  |  |  |  |  |  |  |  |  |  |  |
| 1. Low back |  |  |  |  |  |  |  |  |  |  |  |  |
| 1. One or both hips, legs |  |  |  |  |  |  |  |  |  |  |  |  |
| 1. One or both knees |  |  |  |  |  |  |  |  |  |  |  |  |
| 1. One or both ankles, feet |  |  |  |  |  |  |  |  |  |  |  |  |

We appreciate your collaboration**!**
